# Erosive Pustular Dermatosis of the Scalp: A Clinicopathologic Study of Fifty Cases

**DOI:** 10.3390/dermatopathology8040048

**Published:** 2021-09-23

**Authors:** Andrea Michelerio, Camilla Vassallo, Giacomo Fiandrino, Carlo Francesco Tomasini

**Affiliations:** 1 Dermatology Clinic, Fondazione IRCCS Policlinico San Matteo, 27100 Pavia, Italy; c.vassallo@smatteo.pv.it (C.V.); carlofrancesco.tomasini@unipv.it (C.F.T.); 2 Department of Clinical-Surgical, Diagnostic and Pediatric Sciences, University of Pavia, 27100 Pavia, Italy; 3 Anatomic Pathology Unit, Department of Molecular Medicine, University of Pavia and Fondazione IRCCS Policlinico San Matteo, 27100 Pavia, Italy; g.fiandrino@smatteo.pv.it

**Keywords:** erosive pustular dermatosis, histology, pustular spongiotic infundibular folliculitis, scalp, neutrophilic dermatoses, pyoderma gangrenosum, pathergy

## Abstract

Erosive pustular dermatosis of the scalp (EPDS) is an uncommon, pustular, idiopathic disorder typically occurring on the scalp of the elderly, whose diagnosis requires close clinicopathologic correlations. Recently, the primary histopathologic characteristic of EPDS has been identified in some biopsies from hair-bearing scalp lesions as a sterile, vesiculo-pustule involving the infundibulum of hair follicles. To further delineate the clinicopathologic spectrum of the disease, we led a retrospective study of 50 patients (36 males and 14 females) with a diagnosis of EPDS between 2011 and 2021, reviewing clinical and histopathological data. Androgenetic alopecia was present in 32 patients. Triggering factors were present in 21 patients. The vertex was the most common location; one patient also had leg involvement. Two cases were familial. Disease presentation varied markedly from tiny, erosive, scaly lesions to crusted and hemorrhagic plaques, mimicking pustular pyoderma gangrenosum (PPG). Biopsies of patients with severe androgenetic or total baldness produced specimens showing nonspecific pathologic changes (39/50), while in 11 patients with a hair-bearing scalp histopathologic examination, changes were specific. The clinicopathologic similarities between EPDS and PPG suggest that EPDS should be included in the spectrum of autoinflammatory dermatoses. Clinicians could consider the possibility of associated disorders rather than managing EPDS as a sui generis skin disorder.

## 1. Introduction

Erosive pustular dermatosis of the scalp (EPDS) is a rare pustular, idiopathic, inflammatory condition first described in 1979 by Burton and Pye [1]. The disorder typically occurs on the scalp of elderly males and predisposing factors include androgenetic alopecia or sun-damaged skin and/or a history of scalp trauma. However, some cases of EPDS have been reported in younger individuals [2] or even children [3,4]. This dermatosis may also affect other skin sites, including the face [5,6] and extremities [7].

Clinically, this condition is characterized by recurrent small and barely detectable sterile pustules, erosions and variably thickened grey or yellow-brown crusts. The course is chronic, recurrent and progressive, ultimately leading to scarring alopecia [8]. Topical high-potency corticosteroids (clobetasol propionate 0.05% ointment) are the main line of therapy.

The true disease incidence is unknown, and it is not clear whether EPDS is a rare entity or, more likely, an underdiagnosed condition. Indeed, underdiagnosis is frequent and may be attributed both to clinical features mimicking other conditions and the purported lack of distinctive histologic findings. Recently, the primary histopathologic characteristic of EPDS has been identified in some biopsies from hair-bearing scalp lesions as a sterile, vesiculo-pustule involving the infundibulum of hair follicles, leading to the proposal that the disease should be included in the spectrum of neutrophilic dermatoses [8].

This study aims to further expand our previous clinical and pathological study [8] and reports retrospective assessments of patients with a diagnosis of EPDS to describe the epidemiology, clinical presentation, histopathological features, treatment and outcomes of the disease.

## 2. Materials and Methods

A retrospective study was carried out on 50 patients who had been given a diagnosis of EPDS between 2011 and 2021 at the Dermatology Clinic of the University of Pavia and of the University of Turin (Italy). The diagnosis had been based on close clinicopathologic correlations in all cases. The criteria for the EPDS diagnosis included a variable clinical association of erosions, pustules, scales and crusts on the scalp; negative microbiologic studies; and histopathologic exclusion of any other inflammatory skin disorder.

Epidemiological, clinical, histopathological, therapeutic and follow-up data were recorded, including gender and age, disease duration (months), first clinical diagnoses, localization of the lesions, presence and severity of androgenetic alopecia and/or actinic damage, possible triggering factors, comorbidities and associated local conditions. 

Periodic acid–Schiff, Ziehl–Neelsen, Gram and Giemsa histochemical stains and bacterial and fungal tissue cultures were negative in all cases. A direct immunofluorescence study had also been performed in 3 cases and was negative. All patients had a monthly follow-up until symptom remission, followed by quarterly visits. As this was a retrospective study, no institutional review board or approval of human participants was necessary.

## 3. Results

The clinical data of our EPDS patients are listed in Table 1 and Table 2. A total of 50 patients were enrolled—36 males, age range 28 to 91 (average age, 72; median age, 75), and 14 women, age range 15 to 94 (average age, 68; median age 78)—with a 2.5:1 male-to-female ratio. Patient 46 is the daughter of patient 45. The average duration of the disease at diagnosis was 19 months, ranging from 1 to 240 months.

**Figure 1:**
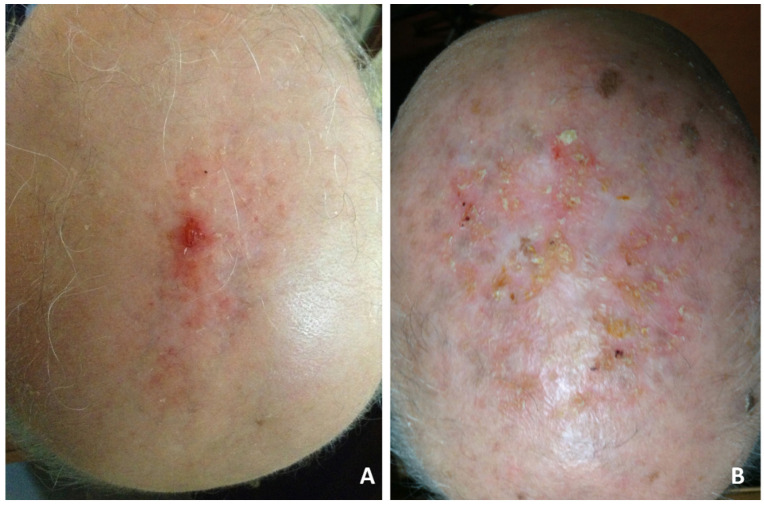
Erosive pustular dermatosis of the scalp; (**A**) solitary eroded and crusting lesions localized on the vertex. Diagnostic consideration was squamous cell carcinoma; (**B**) diffuse scalp involvement of tiny, erythematous, slightly erosive and keratotic papules simulating actinic keratoses.

**Figure 2:**
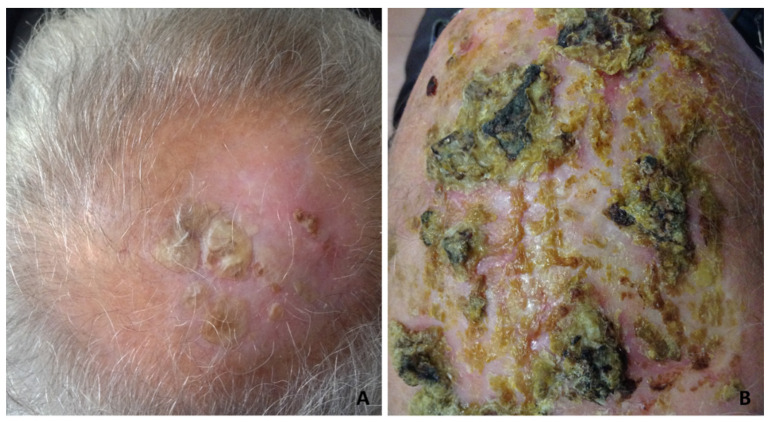
Erosive pustular dermatosis of the scalp; (**A**) thickened keratotic plaques on atrophic skin on the vertex; (**B**) severe clinical presentation with diffuse, thickened, dried yellow crusts, with a greenish or blackish hue resembling pustular pyoderma gangrenosum.

Although the clinical manifestations varied, the most frequent was slightly erythematous erosions and crusts, resembling actinic keratoses (Figure 1A,B), or thickened yellow or yellow-greenish crusts, atrophic skin and scarring alopecia (Figure 2A,B).

**Figure 3:**
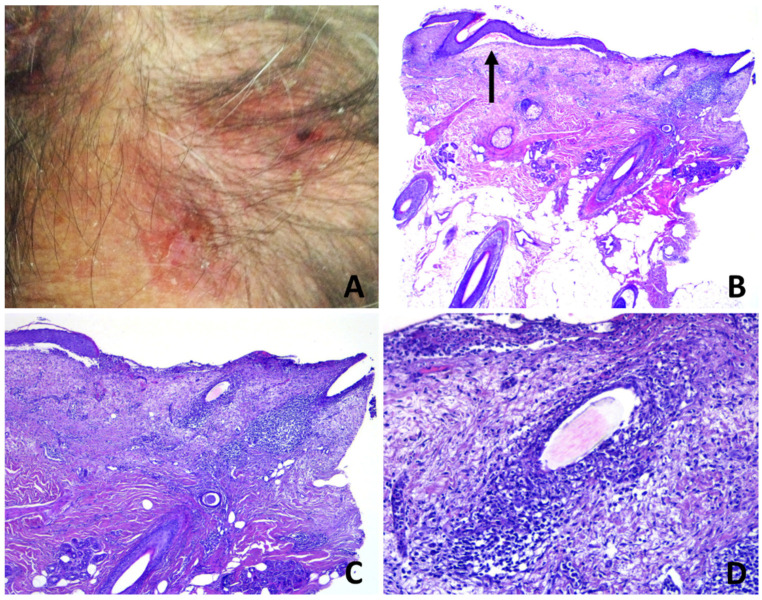
Erosive pustular dermatosis of the scalp; (**A**) erosions and crusts with a few follicular pustules; (**B**) partly eroded skin with underlying superficial folliculitis. A subepidermal clefting is evident (arrow); (**C**) perifollicular and interfollicular fibrosis; (**D**) total destruction of the upper portion of a follicular infundibulum by suppuration with retained hair shaft.

**Figure 4:**
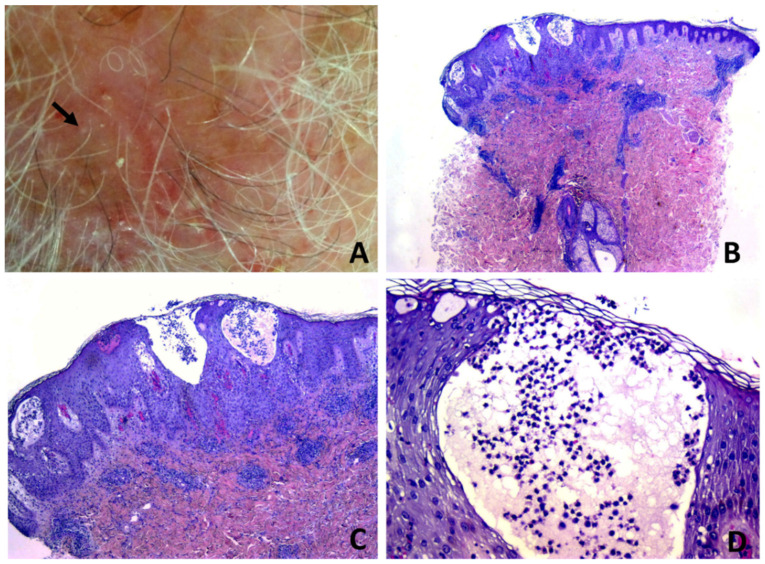
Erosive pustular dermatosis of the scalp; (**A**) follicle-based vesiculo-pustules and erosions; (**B**,**C**) vesiculo-pustules at top of follicular orifices, with sparing of interfollicular epidermis; (**D**) the vesicle contains numerous polymorphonuclear cells.

**Figure 5:**
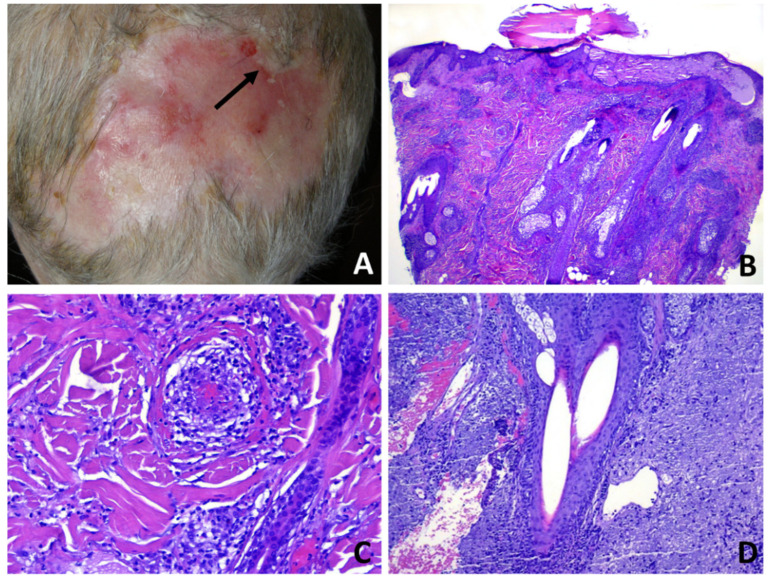
Erosive pustular dermatosis of the scalp; (**A**) scarring alopecia and erosions with presence of follicular pustules, lakes of pus and tufted hairs at the expanding margins (arrow); (**B**) multiple adjacent infundibula showing intrafollicular and perifollicular suppuration; (**C**) perivascular infiltrate of neutrophils with hints of vasculitis. The confluence of multiple vesiculo-pustules at intraepidermal level results into an intraepidermal blister; (**D**) two follicles converge toward a common opening above the entrance of the sebaceous duct.

Pustules were observed in a minority of cases (Figure 3A, Figure 4A and Figure 5A).

The vertex was the most common location (27 patients). The parietal area was involved in five patients, the temporal and the occipital area in three and the frontal area in one patient. A diffuse scalp involvement was present in 14 patients. Notably, one patient had concurrent EPDS lesions on the leg (42) and one on the face (32). The average duration of the disease at diagnosis was 26 months (range: 3–144). Previous mechanical or surgical local trauma was reported in 20 patients, whilst 1 patient reported a previous varicella-zoster infection. Androgenetic alopecia was present in 32 patients (28 males, 4 females) and clinical evidence of actinic damage in 29 patients. 

A total of 46/50 patients were given pre-biopsy clinically specific diagnoses. The most frequently proposed clinical diagnoses were squamous cell carcinoma (SCC) (17 cases) and EPDS (14 patients). Other diagnoses included actinic keratosis (seven cases), basal cell carcinoma (four cases), autoimmune bullous disease (four cases), impetigo (one case), superficial folliculitis (one case), lichen planus pilaris (two cases), frontal fibrosing alopecia (one case), folliculitis decalvans (one case) and Langerhans cell histiocytosis (one case). Ultrapotent topical steroids were effective in almost all patients. Two patients required systemic treatment with corticosteroids and one with tetracyclines to obtain disease control. The average follow-up was two and a half years. 

As to comorbidities, one patient had autoimmune hypothyroidism, one collagenous colitis and one an undifferentiated collagen vascular disease. Two patients had concomitant psoriasis. Moreover, one patient had familiarity for Sjögren’s disease (patient 47) and one for autoimmune thyroiditis (patient 42). 

**Figure 6:**
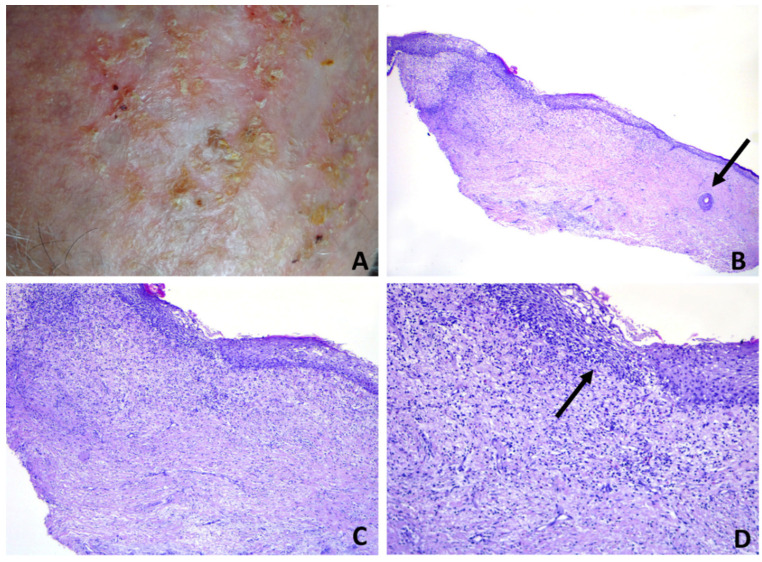
Erosive pustular dermatosis of the scalp; (**A**) tiny erosions, scale crusts and scarring. Pustules are barely detectable; (**B**) diffuse dermal fibrosis. A miniaturized hair follicle at the right lateral margin is seen (arrow); (**C**) dermal inflammatory infiltrate of lymphocytes and neutrophils; (**D**) intraepidermal spongiform pustule (arrow).

**Figure 7:**
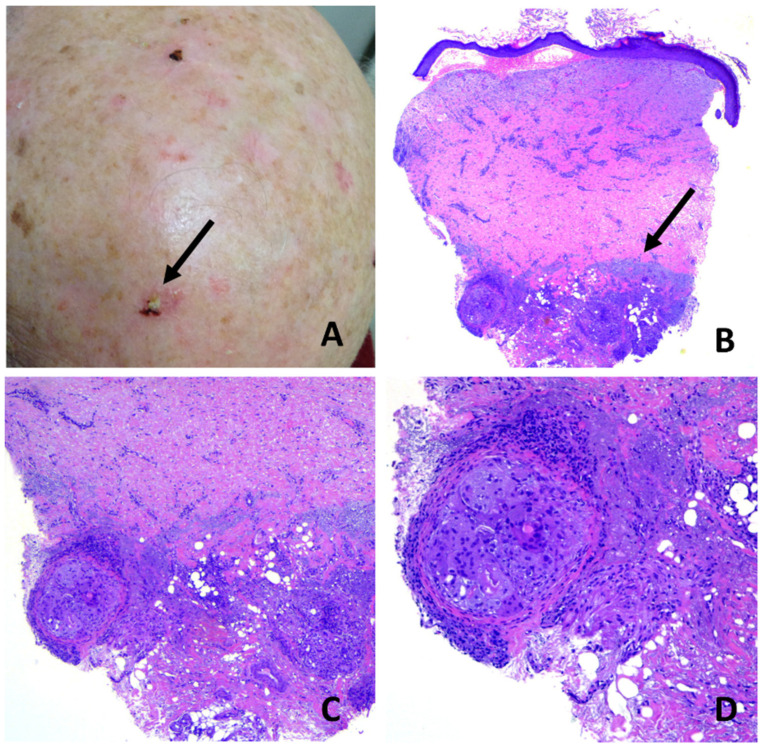
Erosive pustular dermatosis of the scalp; (**A**) chronic, long-standing EPDS with diffuse scarring alopecia. A solitary, erythematous, eroded and crusted plaque was biopsied in the suspicion of squamous cell carcinoma (arrow); (**B**) hemorrhagic blistering in the dermal–epidermal junction, subepidermal clefting and diffuse dermal fibrosis. Note elastotic material pushed down into the deep reticular dermis (arrow); (**C**) focal aggregates of macrophages and foreign-body giant cells containing small hair fragments within deep reticular dermis; (**D**) particular of C.

**Figure 8:**
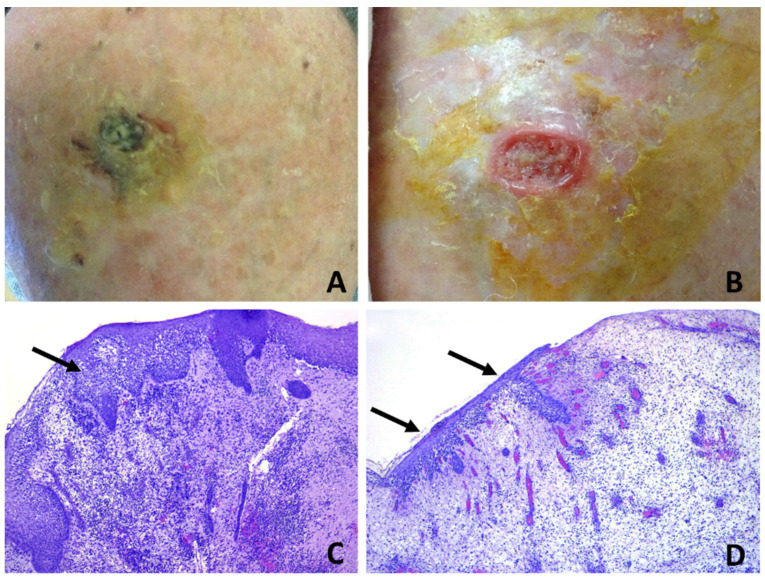
Erosive pustular dermatosis of the scalp; (**A**) black-greenish crusted plaque on the vertex; (**B**) removal of the crust evidences a reddish, raised, undermined border with a purulent granulation base, resembling pyoderma gangrenosum. The lesion was completely excised (**C**) specimen from the margin shows intrafollicular spongiform pustules (arrow); (**D**) specimen from the center evidences erosion with granulation tissue and neutrophilic micro-abscesses within the epithelium of regenerating hair follicles (arrows). The continuing activity of the disease may be inferred by the spongiform pustulation observed in the newly forming hair follicles at the top of the early granulation tissue.

Histopathologically, two types of pathologic changes could be identified: unspecific and specific. Unspecific changes were prevalent and observed in 39/50 cases and were a variable combination of epidermal atrophy with pustulation and dermal scarring (Figure 6B–D), epidermal thickening with subepidermal clefting, scarred dermis and perifollicular granulomas with remnants of hair shafts and multinucleated giant cells (Figure 7B–D) and erosion of the epidermis with foci of pustulation and underlying granulation tissue (Figure 8C,D).

Subepidermal clefting was observed in 11 cases (Figure 3B and 7B). Interestingly, a microscopic examination of 11/50 cases revealed a prominent neutrophilic spongiosis and suppuration involving the infundibular region of the terminal hair follicles, with variable extension to the sebaceous apparatus (Figure 3, Figure 4 and Figure 5). Follicles showed disruption or destruction of the infundibular wall by the inflammatory infiltrate (Figure 3D). Mild extravasation of erythrocytes was observed in 20 cases. Vasculitis was observed in one case (Figure 5C). A variable infiltrate of lymphocytes, macrophages and plasma cells was observed in the surrounding mid and deep reticular dermis, and five cases also had eosinophils. In one case, confluence of spongiotic pustules at the level of infundibular openings had led to vesiculo-bullous pustules within the adjacent epidermis (Figure 5B). Hair tufting was observed in eight cases (Figure 5B,D).

Clinicopathologic correlation showed that infundibular spongiotic pustules were mostly observed in hair-bearing patients, who had mild-to-moderate androgenetic alopecia, whilst nonspecific histopathologic changes were the most common findings in patients with severe androgenetic alopecia or total baldness. Interestingly, in case 7, where a large excision had been performed in the suspicion of SCC, biopsy specimens evidenced different changes according to the various tissue inclusions, ranging from superficial pustular and spongiotic folliculitis (Figure 8C) to eroded skin with granulation tissue and intraepidermal and intrafollicular pustulation (Figure 8D). 

Severe actinic degeneration was observed in 23 patients. Periodic acid–Schiff, Ziehl–Neelson, Gram and Giemsa histochemical stains did not reveal any microorganisms. 

## 4. Discussion

EPDS is a chronic inflammatory dermatosis of unknown etiology that ultimately leads to scarring alopecia. Although still considered a very uncommon entity, dermatologists are having to face this entity with increasing frequency, and EPDS incidence is expected to rise due to improved clinical recognition. 

Although most frequently misdiagnosed as actinic keratosis and/or SCC, there was a clinical suspicion of EPDS in the present study in about 30% of cases, a much higher index than that recorded in our previous study (10%) [8], indicating an increase in the awareness of the disease amongst clinicians. Specific trichoscopic findings, such as severe skin atrophy allowing the visualization of the hair follicles’ bulbs through the epidermidis and enlarged dermal vessels, erosions and crusts, may arise an appropriate index of suspicion and be useful in differentiating EPDS from other scalp disorders associated with scarring alopecia [9]. A high index of suspicion may also positively influence the selection of the biopsy site, increasing the chance of detecting the characteristic changes of the disease, i.e., a vesiculo-pustular infundibulitis with overlying erosion [8]. However, a pathologist may suspect EPDS even if unspecific changes—such as crusted material, eroded epidermis with pustulation and fibrosing and/or granulation tissue—are observed in a histologic specimen from a scalp biopsy carried out for the suspicion of non-melanoma skin cancer and where there is no evidence of malignancy on serial sections. However, a clinicopathological correlation is mandatory in such cases.

EPDS typically affects bald areas of the scalp of elderly individuals but, rarely, young individuals and even children may be affected [2,3,4]. In fact, despite the mean age being higher than that in other studies [9,10], in our study, four patients (42,46,47,50) were under the age of thirty at diagnosis, and, notably, one of them had familiarity (46), suggesting a genetic predisposition. To the best of our knowledge, only one case of familial EPDS has been previously reported [11]. Whilst the male preponderance is recent data [8,12] in the literature, the predilection for the vertex is a consolidated fact [8,9,10,12].

Some studies have reported an association between EPDS and autoimmune diseases [4,10,13,14]. In our series, three patients had concurrent autoimmune systemic diseases, whilst two had a family member affected. Moreover, two patients had psoriasis and one a myelodysplastic syndrome [15]. These data reinforce the hypothesis that EPDS may have a dysimmune pathogenesis and may belong to the spectrum of neutrophilic dermatoses [8,16]. 

The disease evolution in patient 42 provides insights as to the pathogenesis. She was a 23-year-old female who developed EPDS after a mechanical skull trauma, followed by pustular and erosive lesions on the right leg triggered by trauma. EPD of the leg (EPDL) is a poorly understood entity first described by Lanigan and Cotteril in 1987 [17], typically occurring in elderly, mainly female patients and is mainly associated with chronic venous insufficiency and cutaneous atrophy as well as a number of triggering factors [4,18,19,20]. EPDL is not usually associated with analogue lesions on the scalp [20,21]. To the best of our knowledge, this is the first case of concurrent EPDS and EDPL in a young woman. Our patient did not suffer from androgenetic alopecia, venous insufficiency or skin atrophy; thus, the role of minimal trauma was likely pivotal in inducing both the scalp and leg lesions. 

The pathogenesis of EPDS remains obscure. Once triggered, the pathologic process continues with relapses and remissions for years, extending from the primary site to the adjacent skin. The rapid break down of the superficial follicular vesiculo-pustules leads to erosions, followed by crusting and granulation tissue, which ultimately leads to scarring alopecia. This continuous and dynamic process is depicted in Figure 8, where a large excision biopsy had been performed for a suspicion of SCC. The specimens had been taken at different tissue levels of the lesion and showed the presence of intraepidermal/intrafollicular spongiform pustules, both in the intact skin and the eroded and newly forming epidermis above the granulation tissue. It can be assumed that in patients with EPDS, any kind of local trauma, alone or in combination with predisposing factors, is not followed by complete re-epithelization due to local immunological dysregulation with excessive and prolonged neutrophil chemotaxis [22]. Thus, the continuing follicular vesico-pustule formation, that quickly turn into erosions, produces a chronically impaired, wound-healing process where early granulation tissue forms on the surface of the erosion and superimposes on the underlying scar tissue, which gradually leads to scarring alopecia. The scarring process is due to the continuous accumulation of fibrosis in the dermis, which pushes the elastotic collagen into the deep reticular dermis, as evidenced in Figure 7B. The presence of subepidermal clefting observed in some of our casesmay be interpreted as being the result of a faulty restoration of the epidermal basal membrane function, due to a continuous fibrous tissue contraction in an attempt of wound healing and repair. Fibrous tissue contraction may also be responsible for the phenomenon of hair tufting observed in some of our cases.

EPDS is a diagnosis of exclusion; therefore, other conditions, including malignancy, infection, neutrophilic dermatoses and autoimmune blistering disorders, must first be excluded on the basis of clinical, histopathologic, immunologic and microbiologic criteria. Interestingly, pustular pyoderma gangrenosum (PG), a rare subtype of PG, shares some clinical and pathologic features with EPDS, i.e., multiple sterile pustules and erosions and a folliculo-centered neutrophilic infiltrate with infundibular pustules. Indeed, it might be hypothesized that some cases of EPDS may represent a superficial variant of pustular PG [8,23]. Pathergy is also a common finding [24]. PG lesions may occur anywhere on the body, although solitary lesions in atypical locations such as the scalp are uncommon, making this clinical variant especially difficult to diagnose [23]. 

Folliculitis decalvans (FD), sometimes known as tufted folliculitis, is a primary, chronic form of deep neutrophilic folliculitis that usually presents as an expanding patch of alopecia with peripheral follicular pustules and/or tufted hairs on the scalp. It shares with EPDS the typically chronic and relapsing evolution and the predilection for the vertex [9,25]. In contrast to EPDS, infections with bacteria, including *S. aureus*, are frequently observed together with itching and trichodynia [25], only scalp sites with terminal hair follicles are involved, and erosion is not a typical feature. Histopathologically, early FD lesions show prominent keratin plugging of the follicular infundibulum and neutrophilic perifollicular infiltrate. As the follicle ruptures, intrafollicular and perifollicular suppurative and mixed infiltrate can be observed, along with interfollicular fibrosis and follicular tufting. At a later stage, the entire hair follicle is destroyed with granuloma formation and scarring [26].

The clinicopathologic similarities between EPDS and pustular PG are intriguing and further support the proposal that EPDS should be included in the spectrum of autoinflammatory dermatoses, where pathergy plays a pathogenetic role [27]. If confirmed, this hypothesis may have important implications, as clinicians could consider the possibility of associated disorders rather than managing EPDS as a sui generis skin disorder [28].

## 5. Conclusions

In conclusion, EPDS is an enigmatic condition, and its diagnosis depends mainly on the recognition of the evolving clinical features, supported by histopathology. The histopathologic changes depend on the examined lesion, the disease stage and the biopsy site. Although the etiology and exact pathogenesis remain unclear, our study supports the view that EPDS should be included in the spectrum of neutrophilic dermatoses.

## Figures and Tables

**Table 1:** Clinical data of our patients with erosive pustular dermatosis of the scalp.

Case	Sex	Age (y)	Disease Duration (months)	Clinical Diagnosis	Location	Main Clinical Presentation	Aga	Actinic Damage	Traumatic Trigger	Comorbidities/Autoimmune Disorders	Associated Local Skin Condition	Follow-Up (y)
1 *	M	78	10	EPDS	parietal	multiple erythematous, crusted and pustular patches	yes	yes	accidental trauma 16 months earlier	hypothyroidism	/	2
2 *	M	72	12	Langerhans’ cell histiocytosis	vertex	multiple erythematous, crusted and pustular patches	/	/	/	pulmonary Langerhans’ cell histiocytosis	/	5
3 *	M	89	24	AK	diffuse	multiple erythematous, crusted and pustular patches	/	/	accidental trauma 18 years earlier	colon cancer	/	1
4 *	M	66	8	AK	parietal	crusts and erosions	yes	/	/	/	/	2
5 *	M	89	3	AK	diffuse	crusts and erosions	yes	yes	accidental trauma 33 months earlier	/	/	3
6 *	M	75	3	SCC	diffuse	erosions	yes	yes	accidental trauma 57 months earlier	/	AK	2
7 *	F	77	10	SCC	vertex	localized hyperkeratotic nodule	/	/	/	/	/	2
8 *	M	70	20	SCC	vertex	erosions	yes	/	/	/	/	1
9 *	M	75	18	Autoimmune bullous disease	diffuse	scale-crusted lesions	/	/	accidental trauma 6 months earlier	Parkinson’s disease	/	5
10 *	F	69	6	/	parietal	crusts and pustular lesions	yes	yes	accidental trauma 30 months earlier	/	/	2
11 *	M	73	16	SCC	vertex	hyperkeratotic crusted lesions	/	/	multiple accidental traumas	collagenous colitis, prostate cancer, myocardial infarction	/	4
12 *	M	79	20	EPDS	diffuse	crusted lesions	yes	yes	/	prostate cancer	AK and chronic discoid lupus erythematosus	1
13 *	F	78	6	Autoimmune bullous disease	vertex	pustules, crusts and erosions	yes	yes	accidental trauma and previous curettages for NMSC	/	/	2
14 *	F	90	12	SCC	temporal	erythematous crusted nodules and erosions	yes	yes	herpes zoster 12 months earlier	/	/	2
15 *	F	89	24	/	vertex and occipital	crusted lesions	yes	yes	surgery for BCC 24 months earlier	vascular collagen disease	/	4
16 *	M	69	20	BCC	vertex	erosions	yes	yes	skin graft for BCC 4 months earlier	laryngeal cancer	BCC, AK	4
17 *	F	89	20	SCC	occipital	crusted lesions	/	yes	surgery for SCC and AK 16 months earlier	/	AK	5
18 *	M	75	12	AK	vertex	crusted lesions	yes	yes	/	/	/	1
19 *	M	83	4	AK	vertex	crusted lesions	/	yes	/	/	AK	2
20 *	M	82	10	SCC	vertex	erosions	yes	yes	/	/	/	2
21 *	M	84	36	SCC	vertex	crusted lesions	yes	yes	/	prostate cancer, parotid cancer	SCC	5
22 *	M	72	3	EPDS, autoimmune bullous disease	vertex	crusted lesions	yes	yes	/	psoriasis	/	6
23 *	M	63	12	SCC	vertex	erosions	/	/	electrosurgery for NMSC 12 months earlier	dilated cardiomyopathy	BCC	5
24 *	M	80	6	SCC	vertex	crusted lesions	/	/	accidental trauma 30 months years earlier	/	/	4
25 *	F	79	12	BCC	diffuse	erosions	/	/	/	/	/	1
26 *	M	73	12	SCC	parietal	nodular crusted lesion	yes	yes	/	/	/	8
27 *	M	72	36	BCC	diffuse	erosions	yes	/	/	prostate cancer	/	4
28 *	M	76	13	/	diffuse	erosions	yes	/	multiple accidental traumas	/	/	3
29 *	M	77	15	SCC	diffuse	erosions	yes	yes	/	diabetes, chronic obstructive pulmonary disease,	AK	4
30 *	F	80	20	SCC	diffuse	erosions	no	no	/	/	/	5
31	M	70	3	AK, EPDS	diffuse	hyperkeratotic crusted lesions, pustules, erosions	yes	yes	multiple accidental traumas	/	/	1
32	F	94	36	BCC	temporal and face	crusted lesions, erosions, scarring alopecia	no	yes	/	/	AK	2
33	M	72	3	EPDS	diffuse	crusted lesions, erosions	yes	yes	multiple accidental traumas	psoriasis	AK	1
34	M	65	30	frontal fibrosing alopecia	vertex	erosions	yes	yes	/	diabetes, hepatic cirrhosis	SCC	1
35	F	82	14	SCC, EPDS	vertex	crusted lesions, erosions	no	no	/	/	/	2
36	M	81	24	EPDS, AK	vertex	crusted lesions, erosions	yes	yes	/	/	AK	2
37	F	71	7	EPDS	diffuse	crusted lesions	no	no	/	myelodysplastic syndrome	Cutaneous GVHD	1
38	M	66	3	EPSD	vertex	crusted lesions, erosions	yes	yes	scalp skin graft, skull trauma 7 years earlier	/	SCC	1
39	M	77	10	EPDS	vertex	erosions, pustular at periphery	yes	yes	/	prostatic carcinoma	/	2
40	M	84	6	EPDS	diffuse	pustular and pus lakes, crusts	yes	yes	LMM excision 6 months earlier	HCV, hepatic disease, prostatic hypertrophy, secondary platelet deficiency	AK	1
41	M	57	12	EPDS	vertex	crusts, erosions, pustules	yes	yes	sunburns, skull trauma 3 years earlier		AK	4
42	F	23	1	lupus, lichen planus pilaris, folliculitis decalvans	vertex, right leg involved after trauma	Scalp: tufted hairs, intracorneal pustules and crusts. Right leg: intracorneal pustules	no	no	skull trauma one month earlier	/	/	2
43	M	81	6	SCC, EPDS	vertex	multiple crusts	yes	yes	/	/	infiltrating SCC	1
44	M	77	6	SCC, EPDS	vertex	crusts, follicular pustules	yes	yes	/	epilepsy, valvopathy	/	2
45	M	40	240	lichen	temporal, vertex	cicatricial alopecia, crusts, follicular pustules	yes	/	/	/	/	2
46	F	16	36	/	parietal, occipital	crusts, erosions, cicatricial alopecia, tufting	/	/	/	/	/	3
47	F	15	24	impetigo/folliculitis	vertex	crusts	/	/	/	/	/	1
48	M	52	12	seborrheic pemphigus, lichen planopilaris	vertex, frontal	crusts, erosions, pustules, cicatricial alopecia	yes	/	/	/	/	1
49	M	91	18	SCC	vertex	Crusts, granulation tissue	yes	yes	/	/	/	1
50	M	28	48	impetigo/folliculitis	Vertex	Crusts, erosions	/	/	/	/	/	1

* Cases from Tomasini C, Michelerio A. “Erosive pustular dermatosis of the scalp: A neutrophilic folliculitis within the spectrum of neutrophilic dermatoses: A clinicopathologic study of 30 cases”. J Am Acad Dermatol. 2019 Aug; 81(2):527–533, in the same order. EPDS: erosive pustular dermatosis of the scalp; AK: actinic keratosis; F: female; M: male; BCC: basal cell carcinoma; NMSC: non-melanoma skin cancer; SCC: squamous cell carcinoma; y: years; /: not present.

**Table 2:** Clinical data summary.

		n (%)
Age group		
	15–31 years old	4 (8)
	32–48 years old	1 (2)
	49–65 years old	6 (12)
	66–82 years old	30 (60)
	83–100 years old	9 (18)
Gender (M/F)		36 (72)/14 (28)
Average disease duration at diagnosis (months)		19
Involved scalp site		
	Diffuse	14 (28)
	Vertex	27 (54)
	Parietal	5 (10)
	Temporal and occipital	3 (6)
	Frontal	1 (2)
Androgenetic alopecia (M/F)		28 (56)/4 (8)
Actinic damage		29 (58)
Clinical diagnosis		
	Squamous cell carcinoma	17 (34)
	EPDS	14 (28)
	Actinic keratosis	7 (14)
	Basal cell carcinoma	4 (8)
	Autoimmune bullous disease	4 (8)
	Other *	7 (14)
Average follow-up (years)		2.5

* Described in more detail in the results. EPDS: erosive pustular dermatosis of the scalp.

## Data Availability

Data are contained within the article.
